# Surgical Third Nerve Palsy as a Rare Presentation of Tuberculous Meningitis: Early Clinical Judgement and Advanced Imaging

**DOI:** 10.7759/cureus.111014

**Published:** 2026-06-17

**Authors:** Puteri Nur Athirah Megat Mohamad Aminuddin, Mohd Sufian Adenan, Hairuddin Achmad, Fatin Syahirah Nahrowi

**Affiliations:** 1 Internal Medicine, Hospital Kuala Lumpur, Kuala Lumpur, MYS; 2 Neurology, Hospital Kuala Lumpur, Kuala Lumpur, MYS; 3 Radiology, Hospital Kuala Lumpur, Kuala Lumpur, MYS

**Keywords:** anti-tubercular therapy (att), cerebrospinal fluid (csf), contrast enhanced computed tomography (cect), tuberculosis (tb), tuberculous meningitis (tbm)

## Abstract

A 22-year-old man from Bangladesh presented with a subacute febrile illness, a history of frontal headache, neuropsychiatric changes, and a right third nerve palsy (ptosis and mydriasis). Neuroimaging revealed hydrocephalus and multifocal cerebral infarcts. Cerebrospinal fluid (CSF) analysis showed elevated protein with normal glucose; however, all initial microbiological assays, including GeneXpert MTB/RIF (Mycobacterium tuberculosis/rifampicin) and culture, were negative. A clinical diagnosis of tuberculous meningitis (TBM) was made based on the clinical presentation, neuroimaging progression, and epidemiological risk factors. Treatment commenced with anti-tubercular therapy and high-dose steroids. His neurological symptoms markedly improved with medical management, averting the need for neurosurgical intervention for hydrocephalus. This case underscores that TBM can present with an isolated cranial nerve deficit. Therefore, a high clinical index of suspicion is paramount, and treatment should not be delayed.

## Introduction

Tuberculosis (TB) remains a global health burden, with an estimated 10.4 million new cases and 6.3 million notified cases reported in 2016. According to the World Health Organization's Global Tuberculosis Report 2017, TB remains a leading infectious killer worldwide, with an estimated 10.4 million new cases and 1.7 million deaths in 2016. While pulmonary disease is most common, extrapulmonary TB (EPTB) constitutes a significant proportion (16%) of the global caseload. Among EPTB manifestations, central nervous system (CNS) involvement, particularly tuberculous meningitis (TBM), is the most severe, reportedly accounting for 5-10% of EPTB cases and carrying a mortality rate that can exceed 40% in certain regions [1]. Established risk factors include HIV coinfection (associated with a 21-fold higher risk), younger age (a risk factor for severe forms such as TBM), male gender, and comorbidities such as undernutrition, diabetes, and substance use disorders [1]. Socioeconomic determinants include poverty, overcrowding, and poor ventilation [1,2]. In Malaysia, the annual caseload is reportedly between 20,000 and 25,000, with the highest burdens in Selangor and Sabah, and the mortality rate ranges from 8.8% to 11% [2].

TBM, the most severe neurological manifestation of TB, typically arises from the haematogenous spread of bacilli from a primary thoracic focus [3]. Its initial symptoms are non-specific (e.g., fever and malaise), often delaying diagnosis. A hallmark complication of this condition is cranial nerve palsy, primarily affecting the oculomotor nerve (CN III). This is caused by inflammatory encasement and compression of the nerve by thick exudate in the basal cisterns, which can lead to fibrosis and permanent injury [4]. Furthermore, diagnosis is challenging. Only 57% of pulmonary TB cases are bacteriologically confirmed, and no single pathognomonic test for TBM is available [5].

Neuroimaging is crucial because it reveals features such as basal cistern enhancement (indicative of advanced disease) and evidence of vasculitis-induced cerebral infarcts [5]. Despite treatment, TBM has a high mortality rate (approximately 20% within the first month), and half of the survivors experience severe neurological sequelae [6]. Clinical scoring systems (e.g., Lancet and Thwaites) often guide empirical treatment decisions in suspected cases [7,8].

We report a case of TBM presenting with isolated third cranial nerve palsy, highlighting the importance of maintaining a high clinical suspicion and initiating prompt empirical treatment despite negative mycobacterial confirmation.

## Case presentation

A 22-year-old Bangladeshi male construction worker with no significant past medical history was brought to the emergency department by his friends because of acute-onset fever, strange behaviour, and an inability to open his right eye. The patient could not provide further history; his friends noted a four-day history of headache but were unsure about symptoms of pulmonary tuberculosis, travel history, and urinary or upper respiratory tract symptoms.

On examination, he was febrile (39°C) and hypertensive (150/96 mmHg), but not tachycardic (79 bpm) or in respiratory distress. He was pink and not jaundiced. Neurological assessment revealed a complete right third nerve palsy and right ptosis with a non-reactive pupil dilated to 4 mm, compared to the normal left pupil. Fundoscopy showed no papilloedema. Kernig's and Brudzinski's signs were absent. There were no focal motor deficits in the limbs, and no skin lesions were identified.

Initial non-contrast and contrast-enhanced computed tomography (CT) of the brain revealed multiple ill-defined hypodensities in the bilateral basal ganglia, right internal capsule, corpus callosum, right insular cortex, and right temporal lobe, suggesting infarcts. There was also communicating hydrocephalus (Figure 1a-d). No evidence of abnormal leptomeningeal enhancement was observed after contrast administration. However, subtle hyperdensity was noted within the cerebrospinal fluid (CSF) along the right Sylvian fissure (Figure 1a). CT angiography showed no evidence of aneurysm or vascular malformation.

**Figure 1:**
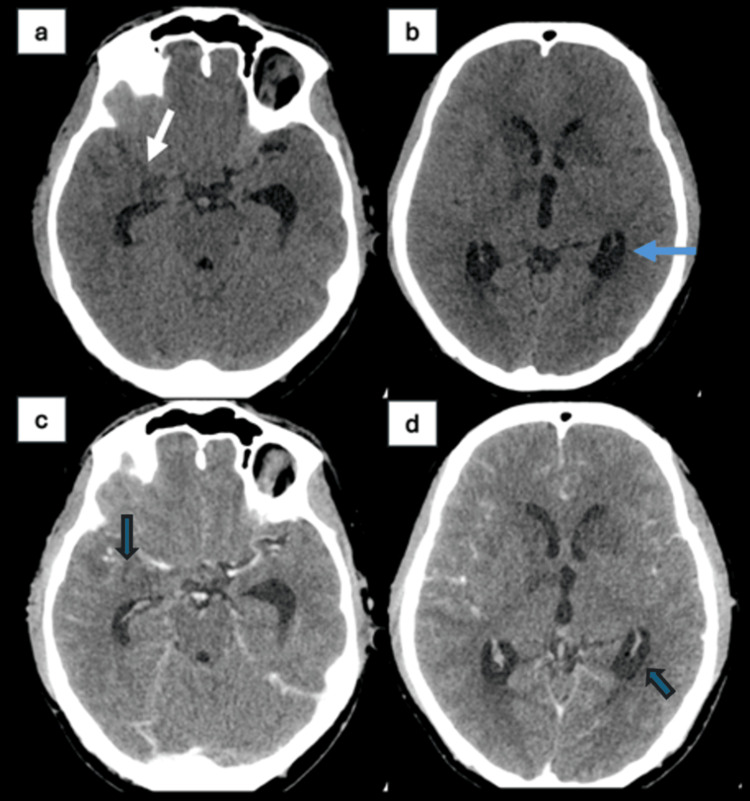
Non-contrast-enhanced CT of the brain in the axial sections (a and b) and contrast-enhanced CT of the brain in the axial sections (c and d) show multiple hypodensities in the bilateral corpus striatum, right internal capsule, and right temporal lobe, consistent with infarcts. Note the subtle density in the right Sylvian fissure (white arrow). Dilatation of the lateral ventricles is noted, particularly in the left lateral ventricle (blue arrow).

Blood investigations revealed an elevated C-reactive protein level of 60 mg/L and a normal white cell count (9.53 × 10^9^/L), platelet count (230 × 10^9^/L), and haemoglobin level (14 g/dL). There was evidence of mild hyponatremia (132 mmol/L), but no evidence of acute kidney injury. Transaminitis was observed with alanine transaminase 118 U/L, alkaline phosphatase (ALP) 61 U/L, total bilirubin 6 mmol/L, and a raised erythrocyte sedimentation rate (80 mm/h). Serum rapid plasma reagin (RPR)/Venereal Disease Research Laboratory (VDRL), hepatitis B surface antigen (HBsAg), hepatitis C virus antibody (HCVAb), and human immunodeficiency virus (HIV) tests were negative.

The patient subsequently underwent lumbar puncture, which demonstrated elevated CSF protein (0.9 g/L) with normal glucose (2.5 mmol/L) and no pleocytosis. Critical microbiological workup, including CSF culture, GeneXpert MTB/RIF, cryptococcal antigen, India ink, acid-fast bacilli (AFB) stain, CSF polymerase chain reaction (PCR) for herpes simplex virus (HSV), and cytology, was negative. Chest radiography was normal. Interferon-gamma release assay (IGRA) (QuantiFERON-TB Gold) and CSF adenosine deaminase (ADA) were not performed because of financial constraints.

While awaiting CSF results, the patient was empirically treated with ceftriaxone 2 g BD and acyclovir according to the meningitis protocol. After three days without clinical response, TBM was suspected based on epidemiological risk factors and imaging findings. Empirical anti-tubercular therapy (ATT) and high-dose intravenous dexamethasone were initiated, while ceftriaxone was continued for broad bacterial coverage.

An MRI of the brain 10 days later confirmed the diagnosis, revealing features characteristic of meningoencephalitis with basal leptomeningeal enhancement complicated by multifocal infarcts, intraventricular exudate, and persistent hydrocephalus (Figure 2).

**Figure 2:**
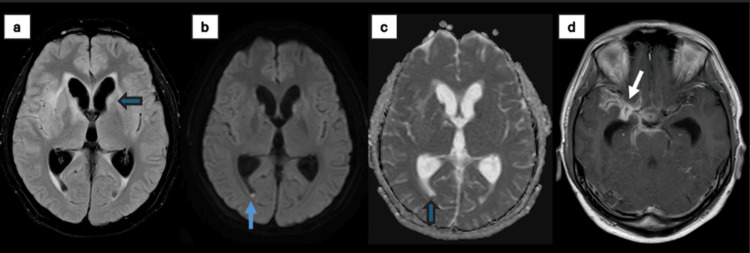
T2-weighted axial MRI of the brain (a) shows hyperintensity in the bilateral basal ganglia and right insular region, with corresponding residual diffusion restriction on axial DWI (b) and ADC (c), and exudate in the dependent portion of the right lateral ventricle (blue arrow). The T1-weighted post-contrast axial image (d) shows diffuse thick leptomeningeal enhancement in the cisterns. Note the thick leptomeningeal enhancement in the right Sylvian fissure, which correlates with the density on prior CT. The ventricles remain dilated in all sequences. MRI: magnetic resonance imaging, DWI: diffusion-weighted imaging, ADC: apparent diffusion coefficient, CT: computed tomography.

The patient showed significant clinical improvement with antitubercular therapy and steroids, with resolution of behavioural changes and improvement in ptosis. A neurosurgical consultation deemed operative intervention unnecessary because of the patient's clinical response. The steroid dose was tapered over eight weeks. The patient returned to Bangladesh to complete his antitubercular treatment, precluding long-term follow-up at our centre.

## Discussion

This case exemplifies the diagnostic challenges of TBM, a condition that often requires treatment initiation based on clinical suspicion without microbiological confirmation. Although TBM is a known cause of cranial nerve palsies, affecting 52% of patients, with the abducens (VI) and oculomotor (III) nerves most frequently involved, an isolated, complete third nerve palsy as the presenting sign is uncommon [9]. The long subarachnoid course of the nerve makes it vulnerable to compression by inflammatory exudates in the basal cisterns, as reported by Gemmani et al. in a case of isolated sixth nerve palsy from a pontine tuberculoma [10]. This presentation requires the urgent exclusion of a posterior communicating artery (PCOM) aneurysm, which was accomplished in this case [11].

In endemic regions, a high index of suspicion for TBM is critical. Our patient's epidemiological risk factors, clinical presentation, and inflammatory CSF profile (elevated protein and normal glucose) justified the immediate initiation of empirical antitubercular therapy and high-dose steroids, a decision supported by the high disability rate (25-78%) associated with TBM. The notoriously low sensitivity of initial CSF tests, including PCR (GeneXpert) and culture, reported at approximately 58-63%, indicates that a negative result cannot rule out the disease. This diagnostic uncertainty underscores the vital role of neuroimaging [12].

In TBM, cranial CT may be normal or may demonstrate abnormalities such as subarachnoid cistern enhancement, sulcal enhancement, hydrocephalus, and tuberculoma. However, in a large retrospective review of 289 TBM patients, Ozates et al. found that CT was normal in only 12% of cases on admission, whereas hydrocephalus was observed in 71% of patients, particularly those with intermediate disease severity [13]. MRI is the superior modality for diagnosis because it provides specific clues, such as basal meningeal enhancement, arteritis, and cerebral infarcts. These findings distinguish TBM, a meningeal disease causing secondary ischaemic injury, from primary parenchymal infections. Imaging not only supports the diagnosis but also directly guides treatment management by highlighting the need for steroids (for vasculitis) or surgical intervention (for obstructive hydrocephalus), emphasising the importance of a multidisciplinary approach [14,15].

Ultimately, this case reinforces that TBM remains a clinical diagnosis. Definitive microbiological confirmation is not always attainable, and treatment should not be delayed when the clinical and radiological picture is compelling. Future studies are required to refine the diagnostic criteria and improve the sensitivity of confirmatory testing.

## Conclusions

Surgical third nerve palsy is an uncommon but critical presenting feature of TBM. This case reinforces that a definitive microbiological diagnosis is not always attainable and should not delay treatment. A high index of clinical suspicion, careful interpretation of neuroimaging, particularly MRI, and prompt initiation of empirical antitubercular and anti-inflammatory therapy are crucial to mitigating the high risk of mortality and severe neurological sequelae associated with this disease.
